# Multidrug-Loaded Lipid Nanoemulsions for the Combinatorial Treatment of Cerebral Cavernous Malformation Disease

**DOI:** 10.3390/biomedicines11020480

**Published:** 2023-02-07

**Authors:** Andrea Perrelli, Annalisa Bozza, Chiara Ferraris, Sara Osella, Andrea Moglia, Silvia Mioletti, Luigi Battaglia, Saverio Francesco Retta

**Affiliations:** 1 Department of Clinical and Biological Sciences, University of Torino, 10043 Orbassano, TO, Italy; 2 CCM Italia Research Network, National Coordination Center at the Department of Clinical and Biological Sciences, University of Torino, 10043 Orbassano, TO, Italy; 3 Department of Pharmacology and Physiology, University of Rochester Medical Center, Rochester, Rochester, NY 14620, USA; 4 Department of Drug Science and Technology, University of Torino, 10125 Torino, TO, Italy; 5 San Giovanni Bosco Hospital, University of Torino, 10154 Torino, TO, Italy; 6 Department of Agricultural, Forest and Food Sciences, University of Torino, 10095 Grugliasco, TO, Italy; 7 Department of Veterinary Sciences, University of Torino, 10095 Grugliasco, TO, Italy; 8 Nanostructured Interfaces and Surfaces (NIS) Interdepartmental Centre, University of Torino, 10124 Torino, TO, Italy

**Keywords:** central nervous system (CNS), cerebrovascular diseases, cerebral cavernous malformation (CCM), KRIT1/CCM1, inflammation, oxidative stress, angiogenesis, nanoemulsions, nanotherapeutics

## Abstract

Cerebral cavernous malformation (CCM) or cavernoma is a major vascular disease of genetic origin, whose main phenotypes occur in the central nervous system, and is currently devoid of pharmacological therapeutic strategies. Cavernomas can remain asymptomatic during a lifetime or manifest with a wide range of symptoms, including recurrent headaches, seizures, strokes, and intracerebral hemorrhages. Loss-of-function mutations in *KRIT1/CCM1* are responsible for more than 50% of all familial cases, and have been clearly shown to affect cellular junctions, redox homeostasis, inflammatory responses, and angiogenesis. In this study, we investigated the therapeutic effects of multidrug-loaded lipid nanoemulsions in rescuing the pathological phenotype of CCM disease. The pro-autophagic rapamycin, antioxidant avenanthramide, and antiangiogenic bevacizumab were loaded into nanoemulsions, with the aim of reducing the major molecular dysfunctions associated with cavernomas. Through Western blot analysis of biomarkers in an in vitro CCM model, we demonstrated that drug-loaded lipid nanoemulsions rescue antioxidant responses, reactivate autophagy, and reduce the effect of pro-angiogenic factors better than the free drugs. Our results show the importance of developing a combinatorial preventive and therapeutic approach to reduce the risk of lesion formation and inhibit or completely revert the multiple hallmarks that characterize the pathogenesis and progression of cavernomas.

## 1. Introduction

Cerebral cavernous malformation (CCM) is a major cerebrovascular disease affecting about 0.5% of the general population [1,2,3]. CCM lesions (also known as cavernous angiomas or cavernomas) appear as clusters of abnormally dilated and leaky capillaries, usually devoid of normal vessel structural components of the human neurovascular unit (NVU), including pericytes and astrocytic foot processes, and characterized by a thin endothelium [4,5]. Diagnosis is commonly made by standard spin-echo magnetic resonance imaging (MRI) [6,7,8]. CCM lesions are mainly localized in the central nervous system (CNS), including the brain and spinal cord, but are also known to affect other organs and tissues, including the retina, skin, and liver. CCM is a disease of genetic origin (OMIM 116860) that may either arise sporadically (sCCM), developing a single lesion, or be inherited as an autosomal dominant condition (fCCM), usually characterized by multiple lesions, with incomplete penetrance and highly variable expressivity [9,10]. CCM lesions range in size from a few millimeters to a few centimeters. CCMs can remain clinically silent for a lifetime, or unpredictably give rise, at any age, to clinical symptoms of varying type and severity, including recurrent headaches, focal neurological deficits, seizures, strokes, and even fatal intracerebral hemorrhages (ICH) [11]. As a human genetic disease, three genes have so far been identified, whose loss-of-function mutations cause CCM disease: *KRIT1* (*CCM1*), *CCM2*, and *CCM3* (*PDCD10*) [10]. In particular, *KRIT1* mutations have been identified as the main cause of fCCM, being responsible for over 50% of all familial cases [12]. However, clinical reports in human cases and several studies in both in vitro and in vivo models have clearly shown that the homozygous loss-of-function mutation of one of the three known CCM genes is not fully sufficient to trigger the cavernoma formation, thus suggesting that additional microenvironmental stressful events, including oxidative and inflammatory insults, significantly contribute to CCM disease onset and progression [13,14,15,16,17,18,19,20,21,22].

Despite the knowledge of different CCM disease phenotypes and pathogenic mechanisms, to date neurosurgical removal of accessible lesions is the only direct therapeutic approach [23]. Therefore, in the past few years, several therapeutic strategies have been tested, aimed at rescuing the major molecular and cellular dysfunctions associated with CCM disease pathogenesis and severity [16]. Most of them are currently emerging, mainly based on compounds endowed with either antioxidant, anti-inflammatory, antiangiogenic, or pro-autophagic properties. Specifically, well-established antioxidant compounds, such as the superoxide dismutase (SOD)-mimetic Tiron [20] and vitamin D [24,25], and well-known autophagy stimulators, including rapamycin (Rapa) and Torin1 [26], have been shown to rescue major molecular dysfunctions associated with cellular models of CCM disease, enhancing mitochondrial function and viability, and significantly decreasing oxidative stress [27,28,29]. Recently, avenanthramides have also been suggested as a potential innovative therapeutic option for CCM disease [19,30,31]. Indeed, the health benefits of natural avenanthramide from oats have been attributed to its antioxidant, anti-inflammatory, and antiproliferative activities, suggesting its potential for prevention and treatment of different human diseases, including cardiovascular and cerebrovascular pathologies, inflammation- and oxidative-stress-related diseases, and cancer [30,31,32].

Therefore, a drug delivery system able to load such therapeutic compounds is needed. Ideally, this would allow overcoming the solubility issues of molecules with different physicochemical features, as well as the engineering of a combination therapy by loading different drugs in the same carrier. In this light, lipid colloidal systems are increasingly used for diagnostic and therapeutic purposes due to their physicochemical and biological properties [33,34]. In particular, liposomes were demonstrated to increase the brain uptake of encapsulated drugs, despite the cumbersome purification methods, the scarce batch-to-batch reproducibility, the limited sterilization procedures, the low drug entrapment, and the poor particle size control, which prevent them from being used for large-scale production [35]. Conversely, lipid-based nanocarriers, such as solid lipid nanoparticles (SLNs) [36] and nanoemulsions (NEs) [37], showed significant technological benefits. They are composed of either solid lipids or oils and stabilized with surfactants. The biocompatible lipid promotes the bioadhesion to the olfactory epithelium, as well as the nanoparticles/nanodroplets partition in the nasal mucosa, while the surfactant acts as a penetration enhancer into the biological membranes. Moreover, these nanoemulsions are cheap to produce, owing to solvent-free and easy-to-scale-up techniques, can encapsulate water-insoluble compounds with a high payload, and are endowed with good physicochemical stability, therefore also suitable for steam sterilization [38]. 

In particular, oil-in-water NEs allow the delivery of compounds with different water solubility and water-in-oil partition coefficients, either in the outer external phase or in the inner core of oil droplets. Within this context, injectable NEs (usually referred to by their commercial trademark Intralipid^®^—ILs) have been used in the last decades as a clinical approach to the parenteral nutrition of patients who cannot eat spontaneously and were successfully commercialized as parenteral drug delivery vehicles [39]. Interestingly, the ILs were shown to be biocompatible with a wide range of cell models and able to incorporate drugs with different water solubility and pharmacological properties, without forming aggregates or precipitates, thus allowing the delivery of combinatorial treatments, which results in improved efficacy and drug dose reduction due to the synergism among drugs and fast cell internalization [40]. Indeed, IL is a nanoemulsion stabilized by soybean lecithin, a water-insoluble surfactant, which is the major constituent of cell membranes, and, therefore, is not associated with cell toxicity, as documented for water–soluble synthetic surfactants [41].

In this experimental study, an NE, suitable for nasal administration, was engineered by loading a drug combination into the IL with the aim of rescuing the major molecular dysfunctions associated with CCM disease phenotypes. Moreover, preliminary biocompatibility and efficacy studies were performed by taking advantage of a well-established cellular model [17,42]. Indeed, both KRIT1-knockout (KRIT1−/−) and KRIT1-overexpressing (KRIT1+/+) mouse embryonic fibroblast (MEF) cell lines were treated with rapamycin (Rapa), yeast avenanthramide I (Avn), and bevacizumab (Bvz), either as a single therapeutic compound or as a combinatorial therapy (herein referred to as Mix), both in the presence and absence of IL as the vehicle. Then, we analyzed through Western blot (WB) analysis the expression levels of molecular biomarkers associated with KRIT1 loss of function, including oxidative-stress-related proteins, such as the transcriptional factor forkhead box class O1 (FoxO1) and its downstream target superoxide dismutase 2 (SOD2); autophagy biomarkers, such as light chain 3 (LC3) and Sequestrosome-1 (p62); and angiogenic factors, such as vascular endothelial growth factor (VEGF) and its main receptor VEGFR2.

## 2. Materials and Methods

### 2.1. Chemicals

We used 10% Intralipid (Fresenius Kabi, Bad Homberg, Germany) as the blank IL. Avastin^®^ was from Roche-Genentech (South San Francisco, CA, USA). Avn was synthesized in-house [32]. Rapa, isopropanol, and trifluoroacetic acid (TFA) were from Alfa-Aesar (Haverhill, MA, USA). Sodium docusate (AOT) was from Merck (Darmstadt, Germany). Polystyrene sulfonate (PS), 60.000–90.000 dextran, and acetonitrile were from Sigma-Aldrich (St. Louis, MO, USA). 

### 2.2. IL Formulation 

IL loaded with a combination of drugs (IL-Mix) was obtained as follows: a stock Rapa-IL was prepared by dissolving 0.9 mg Rapa in 2 mL IL, and 100 μL of stock Rapa-IL was diluted in 0.9 mL blank IL (45 μg/mL, 45 μM Rapa). 

Avn was subsequently solubilized at a final concentration of 1.5 mg/mL (5 mM). Then, 50 uL of AOT 4.5 mg/mL and 40 μL of Avastin^®^ were added (1 mg/mL, 6.6 μM Bvz final concentration), allowing an ion pair at a 1:150 AOT-Bvz molar ratio to be formed [40]. Droplet aggregation was avoided with 50 μL of PS 10 mg/mL as the de-bridging agent.

Control conditions for cell studies were the following: IL + Avn, IL + Rapa, IL + Bvz (obtained by loading the single compounds in IL), free Avn and free Rapa in dimethylsulfoxide (DMSO; 15 mg/mL—50 mM and 4.5 mg/mL—4.5 mM, respectively), and Avastin^®^ (25 mg/mL—160 μM). The combination of the free drugs (Mix) was obtained by mixing the single compounds in the same ratio of IL-Mix.

### 2.3. IL Characterization 

The mean droplet size, polydispersity index (PDI), and Zeta potential of ILs were determined 1 h after formulation by means of dynamic light scattering (DLS; 90 Plus, Brookhaven, NY, USA). Mean size was measured at 90° angle, while Zeta potential at 15°, both at 25 °C. All measurements were performed in triplicate. The homogeneity of the NEs was assessed with optical microscopy (DM2500, Leica Microsystems, Wetzlar, Germany, equipped with a Moticam 410 camera).

Drug recovery (%) was determined by RP-HPLC as the ratio between the actual and the theoretical drug concentrations, after 2-step extraction from the formulations. Briefly, Avn and Rapa were extracted from 50 μL ILs with 100 μL acetonitrile, followed by centrifugation at 14.000× *g* rpm (Allegra 64R centrifuge, Beckman Coulter, Brea, CA, USA). Bvz-AOT ion pair was then extracted from the precipitate with 100 μL acetic acid, followed by addition of 50 μL water, in order to precipitate the lipids by means of 14.000 rpm centrifugation. Since the PS-Bvz-AOT complex overcomes the molecular size limit of RP-HPLC, Bvz-AOT extraction was performed prior to PS addition.

EE%, defined as the ratio between the drug within the lipid droplets and the total drug present in the formulation, was determined in ILs loaded with single therapeutic agents, after dextran gradient centrifugation. Briefly, 0.5 mL IL-Avn and IL-Rapa were diluted with 0.5 mL of 30% 60.000–90.000 dextran and centrifuged at 26.000× *g* rpm (Allegra 64R centrifuge). The pellet was re-suspended in 0.5 mL water, and these purified formulations underwent the same extraction procedure used for recovery (%) quantification. For EE%, instead, Bvz was determined by means of electrophoresis/densitometry. 

### 2.4. HPLC 

HPLC analysis was performed as follows. A YL9100 HPLC system was used, consisting of a YL9110 quaternary pump, a YL9101 vacuum degasser, and a YL9160 PDA detector, linked to a YL-Clarity software for data analysis (Young Lin, Hogyedong, Anyang, Korea). A 300 nm pore size C8 Tecnokroma Tracer Excel 25 × 0.4 cm column was employed, working at 75 °C and with 1 mL/min eluent flow rate. Mobile phases were the following: eluent A (0.1% TFA) and eluent B (70% isopropanol, 20% acetonitrile, 10% water, 0.1% TFA). Gradient was as follows: 0 min—90% A; 15 min—40% A; 20 min—40% A; 21 min—90% A. Detection with PDA was achieved at 220 nm (Bvz), 277 nm (Rapa), and 305 nm (Avn) wavelengths. Retention times were 7.5 min for Avn, 10.9 min for Bvz, and 16.0 min for Rapa.

### 2.5. Electrophoresis

Bevacizumab was quantified by electrophoresis/densitometry: 3 μL of each sample was electrophoresed at 180 V on 12% acrylamide gel for 60 min using a Tris–glycine running buffer. Separated proteins were stained with the silver staining protocol. Lane quantification was performed with ImageJ 1.50i (version 1.50i, University of Wisconsin, Madison, WI, USA) [40].

### 2.6. Cell Culture and Treatment

KRIT1−/− (or KRIT1-knockout) mouse embryonic fibroblast (MEF) cell lines were obtained from KRIT1−/− E8.5 mouse embryos, while KRIT1+/+ (KRIT1-knockin) MEFs were obtained by infecting KRIT1−/− cells with a lentiviral vector encoding KRIT1 [17,42]. Cell culture was performed at 37 °C and 5% CO_2_ in Dulbecco’s modified Eagle’s medium (DMEM; Gibco, Carlsbad, CA, USA) with the supplementation of 10% fetal bovine serum (FBS; Gibco), 1% penicillin/streptomycin antibiotic mixture (100 U/mL; Invitrogen), and 2 mM glutamine (Gibco).

Preliminary viability assays were conducted in KRIT1−/− and KRIT1+/+ MEFs treated for 4, 8, 12, 16, 24 h at 1:100, 1:200, 1:400, 1:500, and 1:1000 dilution in sterile PBS with ILs in order to find the best timeframe and concentration to use as cell treatment. These studies were performed using the Cell Proliferation Kit I (MTT) (#11465007001, Roche, Milwaukee, WI, USA) and a colorimetric assay for the nonradioactive quantification of cellular proliferation, viability, and cytotoxicity, following the manufacturer’s instructions. Measurement of cell proliferation and viability upon treatment was made in 96-well-plate format with wavelengths between 550–600 nm.

In separate experiments, KRIT1−/− and KRIT1+/+ MEFs were treated with NEs loaded with Rapa, Avn, Bvz, or with Mix to a final concentration of: Avn = 15 μg/mL (50 μM), Rapa = 0.46 μg/mL (500 nM), Bvz = 10 μg/mL (70 nM). Then, we analyzed the expression levels of biomarkers of KRIT1 loss of function, including SOD2, VEGF, and autophagy (p62), through WB analysis. Oxidative stress (FoxO1 and SOD) and defective autophagy (p62, LC3) biomarkers were evaluated after 24 h treatment and VEGF expression after 12 h treatment. Indeed, while extracellular VEGF stably decreases upon Bvz exposure, its rapid decrease at intracellular level induces new VEGF expression within 24 h. Therefore, the intracellular VEGF lowering effect can be better appreciated after a shorter exposure to Bvz [43,44]. The results were compared with cells treated with free Rapa, Avn, Bvz, and Mix, at the same concentrations. The given concentrations of compounds were based on the outcomes of previous studies and preliminary data [18,19,26,32].

### 2.7. Protein Extraction and Western Blot

Extraction of total proteins was performed by lysing cells in precooled homemade lysis buffer (RIPA). Protein concentration in cell extracts was determined spectrophotometrically using the Bio-Rad Protein Assay Dye Reagent Concentrate (Bio-Rad Laboratories, Milan, Italy). An equal amount of proteins (30 µg) of total cell extract supernatant for each sample was used for WB analyses. Protein samples were treated with homemade Laemmli buffer, boiled at 95 °C for 5 min, and then resolved on either 10 or 12% homemade polyacrylamide gels for SDS-PAGE. Samples were then blotted onto a nitrocellulose membrane using iBlot Dry Blotting System (Invitrogen, Carlsbad, CA, USA). Subsequently, the incubation of the nitrocellulose membrane with non-fat dry milk 5% in TBS for 1 h at room temperature blocked the unoccupied protein-binding sites. Membranes were then incubated overnight at 4 °C with specific primary antibodies for each biomarker using an appropriate dilution. Membranes were then washed three times with TBS/0.3% Tween-20 (Sigma-Aldrich, Milan, Italy) (TBST) for 5 min each and incubated for 1 h at room temperature with a secondary anti-mouse or anti-rabbit antibody conjugated with the horseradish peroxidase (HRP) (Abcam, San Francisco, CA, USA) diluted 1:5000 in TBST. After three washes of 5 min each with TBST, antigen–antibody complexes were visualized by chemiluminescent detection of peroxidase activity using the ChemiDoc Touch Imaging System (Bio-Rad Laboratories, Milan, Italy). All membranes were re-probed with the antibody for the housekeeping protein α-tubulin as an internal control for protein loading. Protein bands from WB images were quantified by densitometry using the ImageJ software, and their relative amounts were normalized to the levels of α-tubulin.

### 2.8. Antibodies

Primary antibodies used in the present study include the following: rabbit anti-KRIT1 mAb (ab196025), 1:1000 dilution (Abcam, San Francisco, CA, USA); mouse anti-α-tubulin mAb (B-5-1-2), 1:5000 dilution (Sigma-Aldrich, Milan, Italy); rabbit anti-SQSTM1/p62 mAb (D1Q5S), 1:1000 dilution (Cell Signaling, Danvers, MA, USA); mouse anti-SOD2 (2A1) mAb (ab16956), 1:2000 dilution (Abcam); rabbit anti-VEGF pAb (ab46154), 1:1000 dilution (Abcam). Secondary antibodies conjugated with horseradish peroxidase (HRP): goat anti-Rabbit IgG (ab6721), 1:5000 dilution (Abcam); rabbit anti-Mouse IgG (ab6728), 1:5000 dilution (Abcam).

### 2.9. Ethical Statement for Cell Model

KRIT1−/− and KRIT1+/+ mouse embryonic fibroblasts (MEFs) were obtained by Dr. Luca Goitre in the laboratory of Cell Biology, under the supervision of Prof. Saverio Francesco Retta, at the Department of Clinical and Biological Sciences of the University of Torino. Specifically, KRIT1−/− and KRIT1+/+ mouse embryonic fibroblast (MEF) isogenic cell lines were established from KRIT1−/− and KRIT1+/+ E8.5 mouse embryos, respectively, using the 3T3 protocol [45], and were previously described [17,42]. The KRIT1 gene was inactivated by homologous recombination, using a vector obtained by Dr. Luca Goitre, deleting a 631 bp genomic region encompassing 77 nucleotides of the first exon, including the ATG codon, and 554 bp of the upstream 59 untranslated sequence, and replacing this region with a pMC1-Neo-Poly(A) cassette. KRIT1+/+ cells were derived from KRIT1−/− MEFs by lentiviral re-expression of KRIT1 in order to obtain KRIT1-null and KRIT1-expressing MEF cells with uniform genetic backgrounds to be used for comparative molecular and cellular biology studies [17,42]. Specifically, KRIT1−/− MEF cells were infected with a lentiviral vector encoding KRIT1 (pCCLsin.PPT.PGK.KRIT1.Wpre, obtained by Dr. Luca Goitre) to restore KRIT1 expression. Cells were cultured in DMEM (Gibco) containing 10% *v*/*v* heat-inactivated fetal bovine serum (FBS; Gibco), 2 mM glutamine (Gibco), 10 U/mL penicillin (Invitrogen), and 10 µg/mL streptomycin (Invitrogen), at 37 °C and 5% CO_2_.

### 2.10. Ethical Statement for Animals

Animals were bred in standard conditions of temperature (24 °C), humidity (between 40 and 60%), and lighting (12 h of light during day and 12 h of darkness during night), with completely free access to water and food. Animal care and experimental use followed the established guidelines of the European Council Directive 86/609/EEC (24 November 1986) and Recommendation 2007/526/EC (18 June 2007) and were approved by the ethics committee of the University of Torino (Torino, Italy).

### 2.11. Statistical Analysis

Data were generated from three independent experiments and expressed as mean ± standard error (SEM). Statistical significance, determined by Student’s *t*-test, was set at *p* < 0.1.

## 3. Results

### 3.1. Characterization of the IL Formulation as Drug Nanocarriers

The physicochemical characterization of the ILs, both blank and loaded with single therapeutic agents and Mix, is shown in Table 1. The three drugs employed had different molecular weights (MWs), lipophilicity, and water solubility. Indeed, while Bvz is a 150 KDa MW monoclonal antibody, Avn is a small molecule mildly soluble in water with a logarithmic partition coefficient (logP) of ~2 [46,47], and Rapa is a 1000 Da macrocyclic lactone with negligible water solubility (2.6 μg/mL) and a very high logP = 4.3 [48,49]. However, no insoluble crystal was detected by optical microscopy (data not shown). Avn and Rapa were loaded as such, while for Bvz a hydrophobic ion pairing approach was chosen because it was reported to increase its antiangiogenic activity [40]. Notably, all three compounds showed a discrete recovery and EE%, both in combination and as single therapeutic agents. EE% was decreased when therapeutic agents were loaded in combination, probably due to the saturation of the loading capacity into the IL oil droplets. However, no insoluble drug crystal was detected by optical microscopy (Figure S1). Therefore, it can be hypothesized that the drugs were completely dissolved either in the IL water phase or IL oil phase. Hence, the dextran gradient method allowed the correct prediction of drug EE% because it allowed the separation of the IL oil phase, which was layered as a pellet after ultracentrifugation. Of note, absolute % drug recovery in the case of Rapa and Bvz can be slightly underestimated (<70%) because of unwanted aspecific adsorption on plasticware, essential for sample processing [50,51].

**Figure 1:**
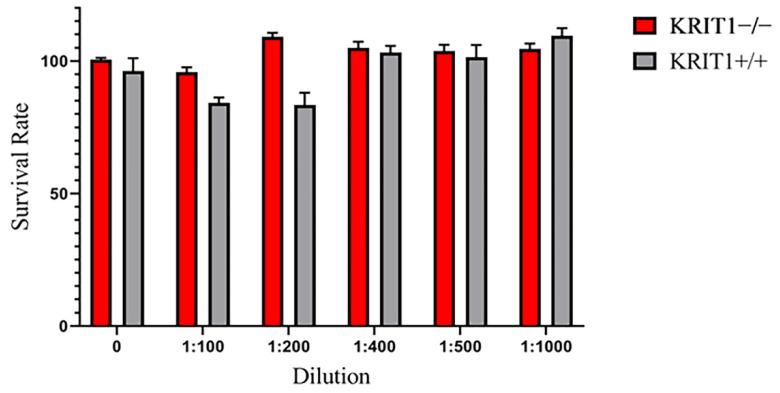
Viability assay performed in KRIT1-knockout (KRIT1−/−) and KRIT1-overexpressing (KRIT1+/+) mouse embryonic fibroblasts (MEFs) left untreated (NT) or treated for 24 h with injectable nanoemulsions (ILs) at different concentrations. After treatment, cell viability was measured as described in Section 2.6 of Materials and Methods, and the best dilution was used to develop the following analyses.

In order to investigate the efficacy of drug-loaded ILs as a combinatorial treatment aimed at rescuing the major molecular dysfunctions associated with CCM disease phenotype, we took advantage of our well-established and previously described cellular model [17,42]. As the first step, we analyzed the toxicological profile of blank lipid NE with specific concentrations (Figure 1) through an MTT viability assay on both KRIT1−/− and KRIT1+/+ MEF cells. This preliminary analysis revealed that IL formulation is perfectly cytocompatible in vitro at any concentration employed, suggesting that it was possible to treat cells at the lowest dilution of 1:100 (Figure 1) in order to maximize the concentration of loaded drugs.

### 3.2. Cell Treatment with Combined Drug-Loaded IL Rescue the Major Molecular Dysfunction Associated with KRIT1 Loss and CCM Disease Phenotype

We next assessed the efficacy of drug-loaded lipid NEs in rescuing the major KRIT1-loss-induced molecular dysfunctions. To this end, we treated either KRIT1−/− and KRIT1+/+ MEF cells using ILs loaded with Rapa, Avn, and Bvz, or Mix, and analyzed the expression levels of biomarkers associated with KRIT1 loss of function. The results of the treatments with free drugs and Mix are shown in Figures S2–S4 (Supplementary Materials).

#### 3.2.1. Rapa- and Avn-Loaded IL Rescue Cellular Antioxidant Response

**Figure 2:**
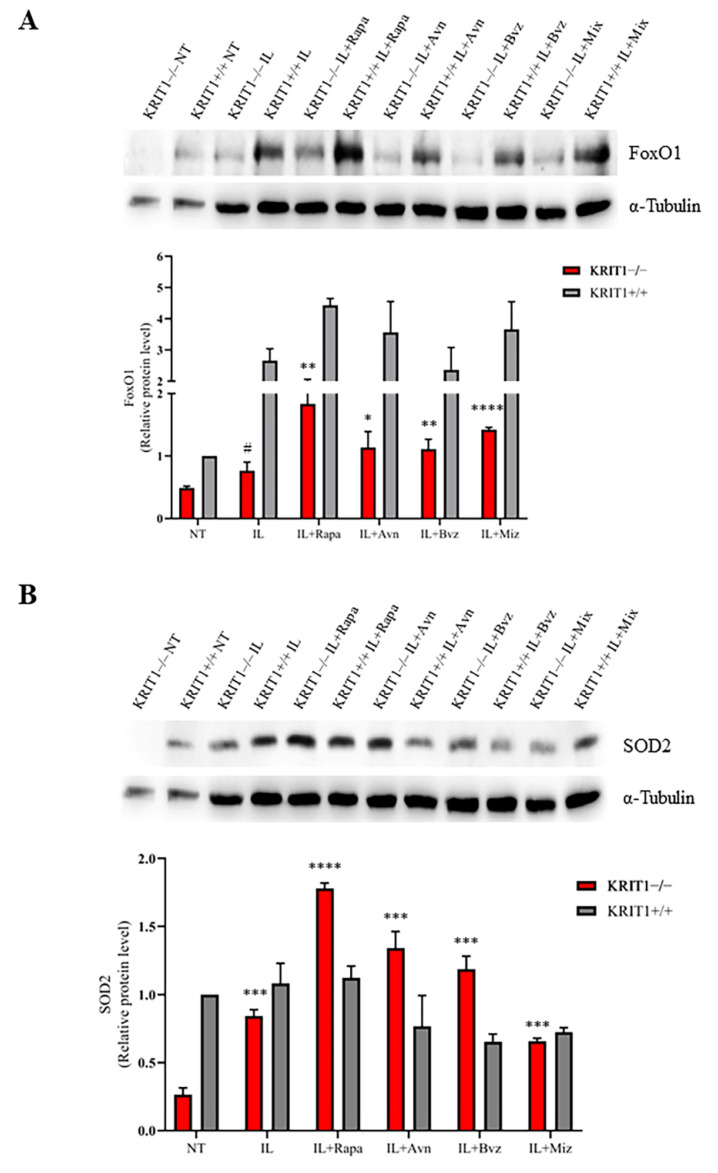
(**A**) Immunoblot analysis and representative histogram of the oxidative stress marker forkhead box class O1 (FoxO1) in KRIT1-knockout (KRIT1−/−) and KRIT1-overexpressing (KRIT1+/+) mouse embryonic fibroblasts (MEFs) untreated (Ctrl) or treated for 24 h with injectable nanoemulsions (ILs) loaded with rapamycin (Rapa), bevacizumab (Bvz), yeast avenanthramide I (Avn), or with a combination of all three compounds (herein referred to as Mix) to a final concentration of: Avn = 15 μg/mL (50 μM), Rapa = 0.46 μg/mL (500 nM), Bvz = 10 μg/mL (70 nM). After treatment, cells were lysed, as described in Materials and Methods, and analyzed for the indicated proteins by Western blot (WB) analysis. (**B**) Immunoblot analysis and histogram representing the quantitative evaluation by densitometric analysis of superoxide dismutase 2 (SOD2) protein expression levels. Quantifications are relative protein level units referring to the average value obtained for KRIT1+/+ samples. α-tubulin was used as internal loading control for WB normalization. Statistical analysis: treatments vs. NT in KRIT−/− MEFs: # *p* < 0.1; * *p* < 0.05; ** *p* > 0.01; *** *p* < 0.005; **** *p* < 0.0001.

Both Rapa- and Avn-loaded lipid NEs are able to increase the expression of FoxO1, both in KRIT+/+ and KRIT−/− cells (Figure 2A), thus reactivating the cell response to oxidative stress and intracellular ROS accumulation due to KRIT1 loss. Moreover, the expression level of the SOD2 enzyme, the main downstream target of FoxO1, is completely restored in KRIT1−/− cells (Figure 2B) and even increased compared to KRIT1+/+ cells upon treatment with drug-loaded lipid nanocarriers, underlining the importance of NE-mediated intracellular delivery as well as the beneficial effect of the loaded compounds, rapamycin and avenanthramide, both endowed with antioxidant properties. Conversely, WB analyses revealed no relevant changes in SOD2 expression levels in KRIT1+/+ cells after treatment. However, the consistent increase in antioxidant biomarkers, also observed in cells treated with blank lipid nanocarriers (Figure 2), suggests that ILs are not completely inert and might play a positive role during treatment, thus contributing to the therapeutic effect of their cargoes.

#### 3.2.2. Rapamycin-Loaded IL Reactivates Autophagy

**Figure 3:**
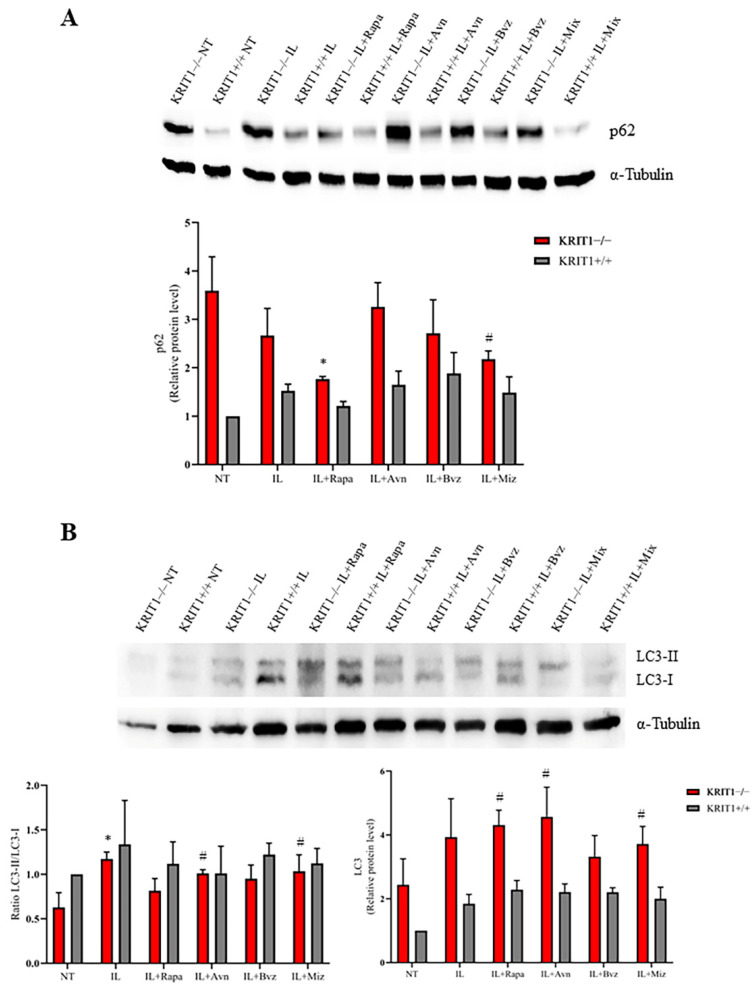
(**A**) Immunoblot analysis and representative histogram of the autophagic marker Sequestosome-1 (p62) in KRIT1-knockout (KRIT1−/−) and KRIT1-overexpressing (KRIT1+/+) mouse embryonic fibroblasts (MEFs) untreated (Ctrl) or treated for 24 h with injectable nanoemulsions (ILs) loaded with rapamycin (Rapa), bevacizumab (Bvz), yeast avenanthramide I (Avn), or with a combination of all three compounds (herein referred to as Mix) to a final concentration of: Avn = 15 μg/mL (50 μM), Rapa = 0.46 μg/mL (500 nM), Bvz = 10 μg/mL (70 nM). After treatment, cells were lysed, as described in Materials and Methods, and analyzed for the indicated proteins by Western blot (WB) analysis. (**B**) Immunoblot analysis and histogram representing the quantitative evaluation by densitometric analysis of light chain 3 (LC3) total protein expression levels and the ratio between LC3-II and LC3-I. Quantifications are relative protein level units referring to the average value obtained for KRIT1+/+ samples. α-tubulin was used as internal loading control for WB normalization. Statistical analysis: treatments vs. NT in KRIT−/− MEFs: # *p* < 0.1; * *p* < 0.05.

Positive effects on KRIT1-loss-dependent defective autophagy were found after cell treatment with different compounds through the analysis of the expression level of the p62 biomarker, which accumulates and is increased when autophagy is inhibited. As shown in Figure 3A, the treatment with Rapa-loaded lipid nanocarriers (Rapa-ILs) decreases p62 protein levels in KRIT1−/− cells to a large extent, and, in a similar manner to the free drug (Figure S3A) [26], it is able to restore the biomarker level of control cells (KRIT1+/+) (Figure 3A). Moreover, the treatment with Rapa-ILs can further increase the level of LC3 II/I (Figure 3B) compared to not-treated (NT) cells, in which the ratio is significantly reduced in KRIT1−/− cells compared to healthy controls (KRIT+/+) due to autophagy inhibition. Indeed, the reactivation of autophagy enhances the production of the microtubule-associated protein 1A/1B-light chain 3 (LC3), an important component of autophagosome membranes, which is usually reduced during autophagy inhibition. Notably, Rapa is able to increase not only the total LC3 level but also the LC3 II/I ratio by the cleavage and conjugation to phosphatidylethanolamine of the inactive LC3-I isoform into the active LC3-II, which plays a key role in the formation of autolysosomes [26]. However, while Rapa can also increase the cellular antioxidant responses through autophagy reactivation (Figure 2), thus allowing the removal of ROS-generating dysfunctional mitochondria, Avn is devoid of a specific pro-autophagic activity.

#### 3.2.3. Bvz-Loaded ILs Partially Reduce Vascular Growth Factor Signaling Pathways

**Figure 4:**
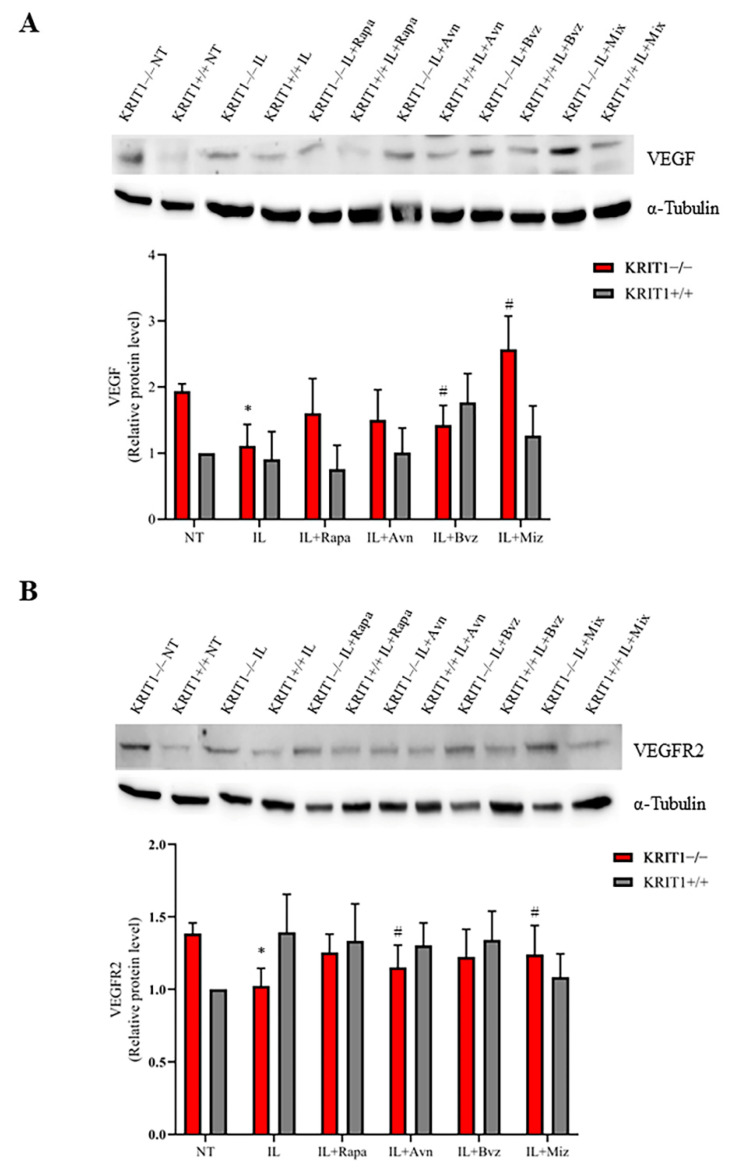
(**A**) Immunoblot analysis and representative histogram of vascular endothelial growth factor (VEGF) in KRIT1-knockout (KRIT1−/−) and KRIT1-overexpressing (KRIT1+/+) mouse embryonic fibroblasts (MEFs) untreated (Ctrl) or treated for 12 h with injectable nanoemulsions (ILs) loaded with rapamycin (Rapa), bevacizumab (Bvz), yeast avenanthramide I (Avn), or with a mixture of all three compounds (herein referred to as Mix) to a final concentration of: Avn = 15 μg/mL (50 μM), Rapa = 0.46 μg/mL (500 nM), Bvz = 10 μg/mL (70 nM). After treatment, cells were lysed, as described in Materials and Methods, and analyzed for the indicated proteins by Western blot (WB) analysis. (**B**) Immunoblot analysis and histogram representing the quantitative evaluation by densitometric analysis of VEGFR2 protein expression levels. Quantifications are relative protein level units referring to the average value obtained for KRIT1+/+ samples. α-tubulin was used as internal loading control for WB normalization. Statistical analysis: treatments vs. NT in KRIT−/− MEFs: # *p* < 0.1; * *p* < 0.05.

A clear upregulation of VEGF and VEGFR2 is found in KRIT1−/− MEFs compared to the healthy controls (KRIT+/+) (Figure 4). Contrary to the promising efficacy of bevacizumab, used as free drug (Figure S3), a small reduction in the expression level of VEGF (Figure 4A) and its main receptor VEGFR2 (Figure 4B) is found in cells treated with NEs loaded with the same chemotherapeutic compounds. Indeed, besides a regulatory effect on VEGF expression levels, several cellular mechanisms can contribute to this particular effect, and ILs might promote the internalization of the high-molecular-weight antibody Bvz, leading to an intracellular sequestration of VEGF.

## 4. Discussion

To date, there are no pharmacological therapeutic approaches for CCM disease. Indeed, given the large heterogeneity and unpredictability of CCM disease onset and progression, as well as the variability of its clinical features, including CCM lesion size, number and localization, and the critical involvement of additional pathological determinants besides mutations in CCM genes, such as stress events, CCM remains one of the major cerebrovascular diseases devoid so far of any preventive and therapeutic approach.

Within this context, some general considerations can help to select specific molecular therapeutic targets for a potential combinatorial therapy. Indeed, several studies in the past decade demonstrated that defective autophagy, ROS accumulation, and increased oxidative stress, and inflammatory responses are associated with CCM gene loss-of-function mutations in both in vitro and in vivo disease models, and fCCM patients [17,18,26], and may justify the high interindividual variability in CCM disease onset and severity [13,14,16,20,32]. Furthermore, abnormal angiogenesis, which is known to be closely associated with oxidative stress and inflammatory responses, represents another major hallmark of CCM lesion development [52,53]. In this light, specific antioxidant and anti-inflammatory nanosystems may represent a promising therapeutic strategy for CCM treatment [13,16,28,54]. Accordingly, whereas the efficacy of combinatorial therapies has been clearly demonstrated [55,56,57,58,59], the multiple targeting of oxidative stress and defective autophagy, by a composite nanosystem endowed with both antioxidant and pro-autophagic activities, has recently emerged as a potential nanomedicine approach for the effective treatment of CCM disease [28].

**Figure 5:**
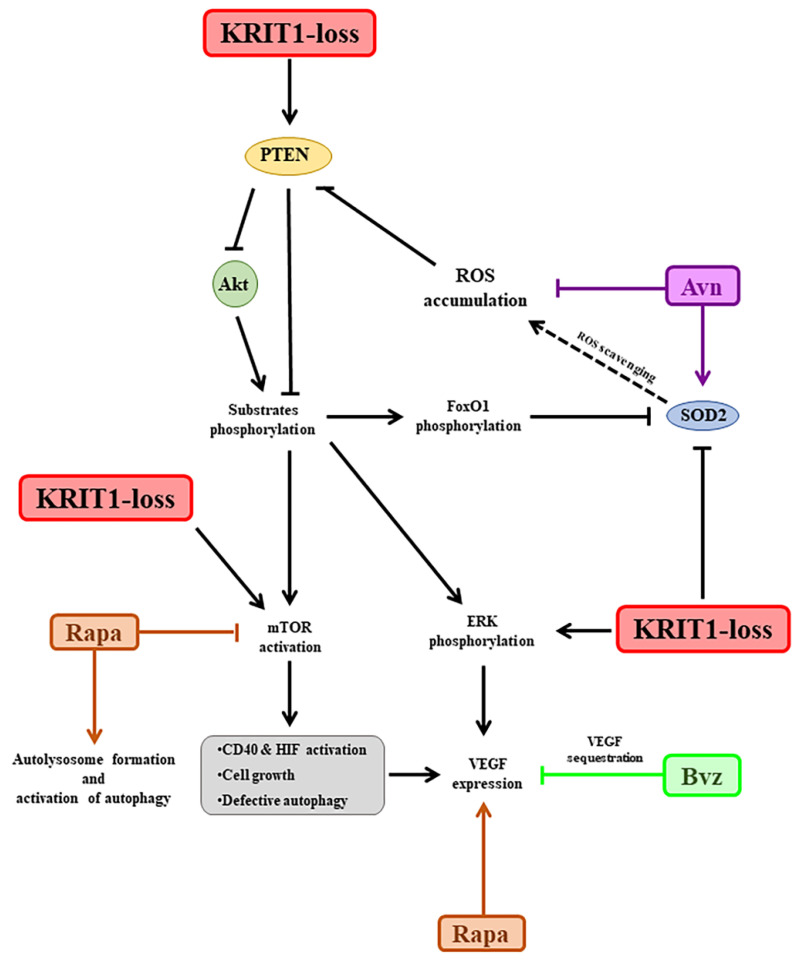
Schematic representation of the molecular targets considered in order to develop the proposed nanomedicine-based combinatorial therapeutic approach for CCM disease. The most significant results have been obtained by targeting the oxidative stress with lipid nanoemulsions containing avenanthramide, both alone and in combination with rapamycin. Conversely, the efficacy of rapamycin as an autophagy promoter needs to be improved in the nanoemulsion, and the effect of the bevacizumab-loaded nanoemulsion in rescuing the abnormal angiogenesis looks contradictory, probably due to the in vitro model employed. Abbreviations: Avn: yeast avenanthramide I; Akt: protein kinase B; Bvz: bevacizumab; CCM: cerebral cavernous malformation; CD40: cluster of differentiation 40; ERK: extracellular-signal-regulated kinase; FoxO1: forkhead box class O1; HIF: hypoxia-induced factor; KRIT1: Krev interaction trapped protein 1; mTOR: mammalian target of rapamycin; PTEN: phosphatase and tensin homolog; Rapa: rapamycin; ROS: reactive oxygen species; SOD2: superoxide dismutase 2; VEGF: vascular endothelial growth factor.

KRIT1 loss causes a significant downregulation of FoxO1 expression and transcriptional activity in CCM disease models [17,60], which has been correlated with the modulation of major cellular stress response regulators, such as protein kinase B (PKB/Akt) and 5′-adenosine monophosphate-activated protein kinase (AMPK) [61,62]. In particular, the significant downregulation of FoxO1 protein levels, detected in the KRIT1−/− cells compared to healthy controls, is followed by a drastic reduction in its downstream target SOD2, a major antioxidant enzyme, which is known to cause a compromised cellular response to oxidative stress [17]. This alteration, together with the upregulation of the nuclear factor erythroid 2-related factor 2 (Nrf2) pathway and glyoxalase system (GLO1 and GLO2), leads to chronic adaptive homeostasis that sensitizes cells to additional stress events [20]. Furthermore, these changes are known to play a role in the oxidative post-translational modifications (PTMs), including the S-glutathionylation of chaperonins, metabolic enzymes, and structural proteins [54,63], underlying the fine-tuned regulation of KRIT1 activity in response to inflammatory and oxidative stress conditions [20,54,63,64]. Interestingly, the signaling pathways involving Akt and AMPK are highly interconnected, sensitive to the cellular metabolic and redox state, and involved in the regulation of autophagy [65] and angiogenesis [66]. This suggests a potential relationship with the pleiotropic effects of altered redox homeostasis and signaling, so far associated with KRIT1 loss of function [13,15,16]. In particular, there is evidence that oxidative inactivation of the phosphatase and tensin homolog (PTEN) protein, a major negative regulator of PI3K-dependent Akt through the dephosphorylation signaling pathway, causes a hyperactivation of Akt and consequent modulation of its downstream targets, including the activation of mammalian target of rapamycin (mTOR) and the inhibition of FoxO1, leading to defective autophagy and enhanced cell sensitivity to oxidative stress [67,68]. Indeed, sporadic CCM lesions are often characterized by causative mutations in genes other than CCM ones [15,69,70] and including PTEN, whose deficiency results in a severe phenotype [62,71]. Furthermore, the CCM phenotype is typically associated with increased VEGF, which is due to the overexpression of both β-catenin [72,73,74] and extracellular-signal-regulated kinase (ERK) [75]. In fact, the Akt phosphorylation pathway, which is significantly enhanced in KRIT1−/− in vitro models [62,75], also leads to the activation of ERK [62,75] and mTOR pathways [26]. In turn, mTOR activates the cluster of differentiation 40 (CD40) [76] and hypoxia-induced factor (HIF) [77] pathways, leading to the upregulation of VEGF (Figure 5). In this light, a treatment based upon antioxidant compounds might allow the restoration of proper cellular antioxidant responses able to reduce the intracellular accumulation of ROS as well as to reactivate the autophagic processes and reduce the sensitization to additional stress events in a cellular model [20,64]. Thus, in our approach, while the treatment with antioxidant Avn should counteract the oxidative stress [31,32], Rapa should reactivate the defective autophagy [26,28,78] and Bvz reduce the activation and function of VEGF and VEGFR2, which might result in the inhibition of angiogenesis [75] (Figure 5).

In our experiments, the effects of drug-loaded ILs were estimated on the main CCM molecular dysfunctions by comparing the mean expression levels of specific biomarkers (Table 2). Consistent with previous results [28,79,80], here we show that IL-Mix has a therapeutic effect on the expression levels of most KRIT1-loss-dependent biomarkers underlying the pathogenesis of CCM disease, with the best results in the FoxO1/SOD2 pathway where a complete reversion of the phenotype is achieved. Indeed, ILs might have a slight antioxidant activity within the intracellular environment, increasing FoxO1 and the antioxidant enzyme SOD2, which contributes to the therapeutic effect of Rapa- and Avn-loaded ILs on the FoxO1/SOD2 axis (Figure 2). Moreover, Rapa-loaded ILs are able to reactivate autophagy, thus reducing the accumulation of p62 and increasing the conversion of LC3-I into LC3-II. However, conflicting results were obtained about the angiogenesis biomarkers, since the promising downregulation obtained after treatments with free drugs was only partially reproduced with Bvz-loaded ILs. We hypothesize that the internalization of IL loaded with Bvz might cause VEGF sequestration inside the cell, thus also reducing its activity on VEGFR2. However, although drug release from ILs obtained in vivo could overcome this issue, further studies are needed to understand in detail the effect that Bvz plays on VEGF and VEGFR2 expression levels in our in vitro model. Indeed, while there is clear evidence for a major role of VEGF signaling in both in vitro and in vivo models of CCM disease [62,72,73], little is known about the potential efficacy of Bvz for cavernoma treatment.

Although the analysis of the biomarkers for oxidative stress (FoxO1 and SOD2) provided reasonable and consistent positive results, validating the fundamental role of this event in contributing to CCM disease pathogenesis [13,16], this study needs further improvements to the analysis of these two sets of therapeutic targets. Indeed, Western blots for the autophagic biomarkers (p62 and LC3 II/I) produced results with a lower degree of significance, probably due to the complex mechanism underlying this process, even if they confirmed the effect of the autophagy promoter rapamycin, especially on the expression level of p62 (Figure 3A) [26,28], while the analysis of biomarkers involved in angiogenesis provided more conflicting data.

Indeed, although we observed a positive trend and a good efficacy of the combined pharmacological approach in this preliminary in vitro study, additional molecular biomarkers unbiasedly identified through genome-wide omics studies [81,82,83,84,85,86,87,88] should be analyzed in future in vitro and in vivo studies in order to validate this innovative combined therapeutic approach.

Nonetheless, these promising results pave the way to an innovative preventive and/or therapeutic approach for CCM disease, with the specific aim of reducing the risk of new lesion formations, decreasing the size of already existing cavernomas, and trying to inhibit or rescue the multiple molecular dysfunctions that characterize its pathogenesis. In this light, it should be pointed out that a therapeutic strategy that allows overcoming the blood–brain barrier (BBB) and the delivery of drugs specifically to the CNS—minimizing their accumulation in non-target tissues and consequent off-target toxicity—represents a fundamental challenge against CCM disease. In particular, the nose-to-brain delivery is a non-invasive method which allows to overcome the BBB, with high patient compliance, a fast onset of action, and a significant reduction in side effects, even if limited by low administration volumes and nasal mucociliary clearance [89]. We recently investigated the nose-to-brain delivery of ILs [90] because the biocompatible lipid component of NEs could allow bioadhesion to the olfactory epithelium and the partition of the nanodroplets in the nasal mucosa, and the surfactant could enhance the penetration of biological membranes [91]. In this light, nose-to-brain delivery of IL-Mix would represent a step forward as an effective precision medicine [37,92,93,94]. Such an approach—which has already been proposed for the commonest cerebrovascular diseases [95,96,97] and, given the emerging molecular links between cerebrovascular and neurological diseases [98,99], for the related neurological co-morbidities [91]—could now be exploited for a rare disease, such as CCM. Overall, the outcomes of these in vitro studies should stimulate further implementation in CCM animal models.

## 5. Conclusions

Lipid NEs have emerged as a promising tool for drug delivery to target multifactorial and complex diseases. Loading drug combinations in a single nanosystem can have the advantages of beneficially merging the pharmacological profiles of drugs, reducing their therapeutic dose with a consequent decrease in off-target toxicity, and optimizing drug delivery. Here, we engineered a multifunctional lipid NE by loading a drug combination with antioxidant, pro-autophagic, and antiangiogenic activity into ILs. Through an established CCM cellular model, we demonstrated the superior ability of multifunctional ILs in rescuing the major molecular dysfunctions of the CCM disease phenotype. Owing to their technological and biological features, the nose-to-brain delivery of such NEs would allow the delivery of different loaded pharmacological compounds directly into the lesion area and would be suitable for chronic treatment. Although further in vivo studies in CCM animal models must still be performed, our results pave the way to an innovative preventive and/or therapeutic treatment for CCM disease, with the specific aim of reducing the risk of onset and the size of cavernomas.

## Figures and Tables

**Table 1:** Physicochemical characterization of blank and drug-loaded ILs. Abbreviations: Avn: yeast avenanthramide I; Bvz: bevacizumab; EE%: entrapment efficiency; IL: injectable nanoemulsion; Mix: yeast avenanthramide I, bevacizumab, rapamycin combination; N.D.: not determined; Rapa: rapamycin. Values were generated from independent experiments and expressed as mean ± standard error (SEM).

	IL-Avn	IL-Rapa	IL-Bvz	IL-Mix	Blank IL
Mean size (nm)	246.0 ± 2.5	250.1 ± 1.8	249.4 ± 1.9	253.5 ± 3.6	277.8 ± 2.5
Polydispersity	0.104	0.079	0.047	0.079	0.183
Z Potential	−37.97 ± 1.10	−40.52 ± 1.10	−35.95 ± 2.06	−44.90 ± 6.81	−39.72 ± 1.94
Drug recovery (%)	98.9 ± 7.3%	66.0 ± 6.1%	76.5 ± 15.7%	Avn: 75 ± 3.5% Bvz: 70.0 ± 17.4% Rapa: 61.7 ± 1.3%	—
Drug EE %	59 ± 6.0%	70 ± 7.0%	33.5 ± 4.9%	Avn: 29.1 ± 1.5% Bvz: 27.6 ± 3.9% Rapa: 67.0 ± 4.9%	—

**Table 2:** Effects of Mix and IL-Mix on the main CCM molecular phenotypes. Abbreviations: Avn: yest avenanthramide I; Bvz: bevacizumab; CCM: cerebral cavernous malformation; NT: not-treated control; FoxO1: forkhead box class O-1; IL: injectable nanoemulsion; LC3-II: light chain 3-II; Mix: yeast avenanthramide I, bevacizumab, rapamycin combination; N.D.: not determined; NT: untreated; p62: Sequestosome-1; Rapa: rapamycin; SOD2: superoxide dismutase 2; VEGF: vascular endothelial growth factor; VEGFR2: vascular endothelial growth factor receptor 2.

		NT	Mix		IL-Mix	
	Parameter	Pathological Condition in CCM	Therapeutic Effect	Rescue of Molecular Dysfunction	Therapeutic Effect	Rescue of Molecular Dysfunction
	Comparison	Δ: NT KRIT1−/− vs. NT KRIT+/+	Δ: Mix vs. NT KRIT1−/−	=: Mix in KRIT1−/− vs. NT KRIT1+/+	Δ: IL-Mix vs. NT KRIT1−/−	=: IL-Mix in KRIT1−/− vs. NT KRIT1+/+
Altered antioxidant responses	FoxO1	Reduced	Yes	Partial	Yes	Complete
	SOD2	Consistently reduced	Yes	Partial	Yes	Complete
Defective autophagy	p62	Increased	Yes	Partial	Yes	Partial
	LC3-II	Increased	Yes	N.D.	Yes	N.D.
Enhanced angiogenesis	VEGF	Increased	Yes	Complete	No	No
	VEGFR2	Increased	Yes	Complete	No	No

## Data Availability

Not applicable.

## Supplementary Materials

The following supporting information can be downloaded at: https://www.mdpi.com/article/10.3390/biomedicines11020480/s1.
