# Supplementary material for: Multidrug-Loaded Lipid Nanoemulsions for the Combinatorial Treatment of Cerebral Cavernous Malformation Disease
Source: Biomedicines. 2023 Feb 7;11(2):480. doi: 10.3390/biomedicines11020480 (PMC9953270; doi:10.3390/biomedicines11020480)
Supplement: Supplementary file 1 [file biomedicines-11-00480-s001.zip › biomedicines-2060495-supplementary.pdf]

Supplementary Materials: Multidrug-Loaded Lipid Nanoemulsions for the Combinatorial Treatment of Cerebral Cavernous Malformation Disease

Andrea Perrelli, Annalisa Bozza, Chiara Ferraris, Sara Osella, Andrea Moglia, Silvia Mioletti, Luigi Battaglia, Saverio Francesco Retta

Figure S1 **S2**

Figure S2 **S3**

Figure S3 **S4**

Figure S4 **S5**

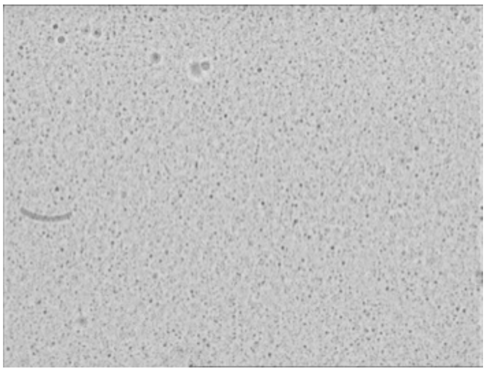

A: IL

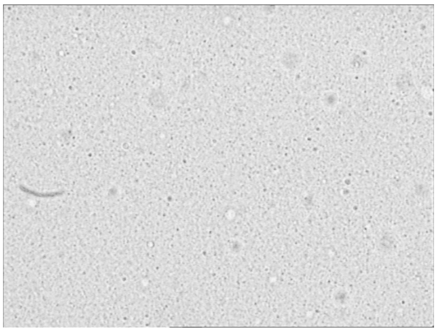

B: IL+Rapa

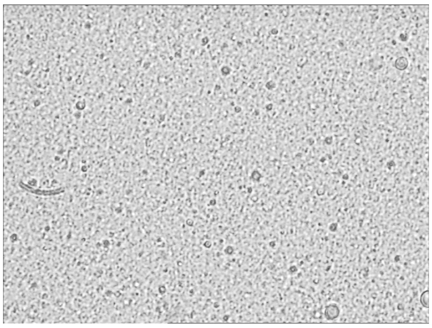

C: IL+Avn

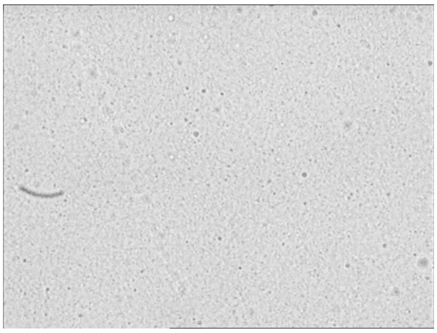

D: IL+Bvz

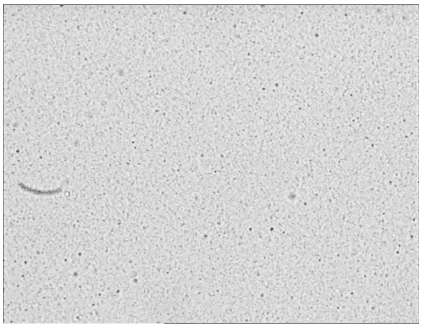

E: IL+Mix

Figure S1. Images from optical microscopy of the formulations under study (630X magnification). A) IL (Intralipid® 10%); B) IL+Rapa (Rapamycin loaded IL); C) IL+Avn (Avenanthramide loaded IL); D) IL+Bvz (Bevacizumab loaded IL); E) IL+Mix (IL loaded with drug combination: Avn, Rapa, Bvz).

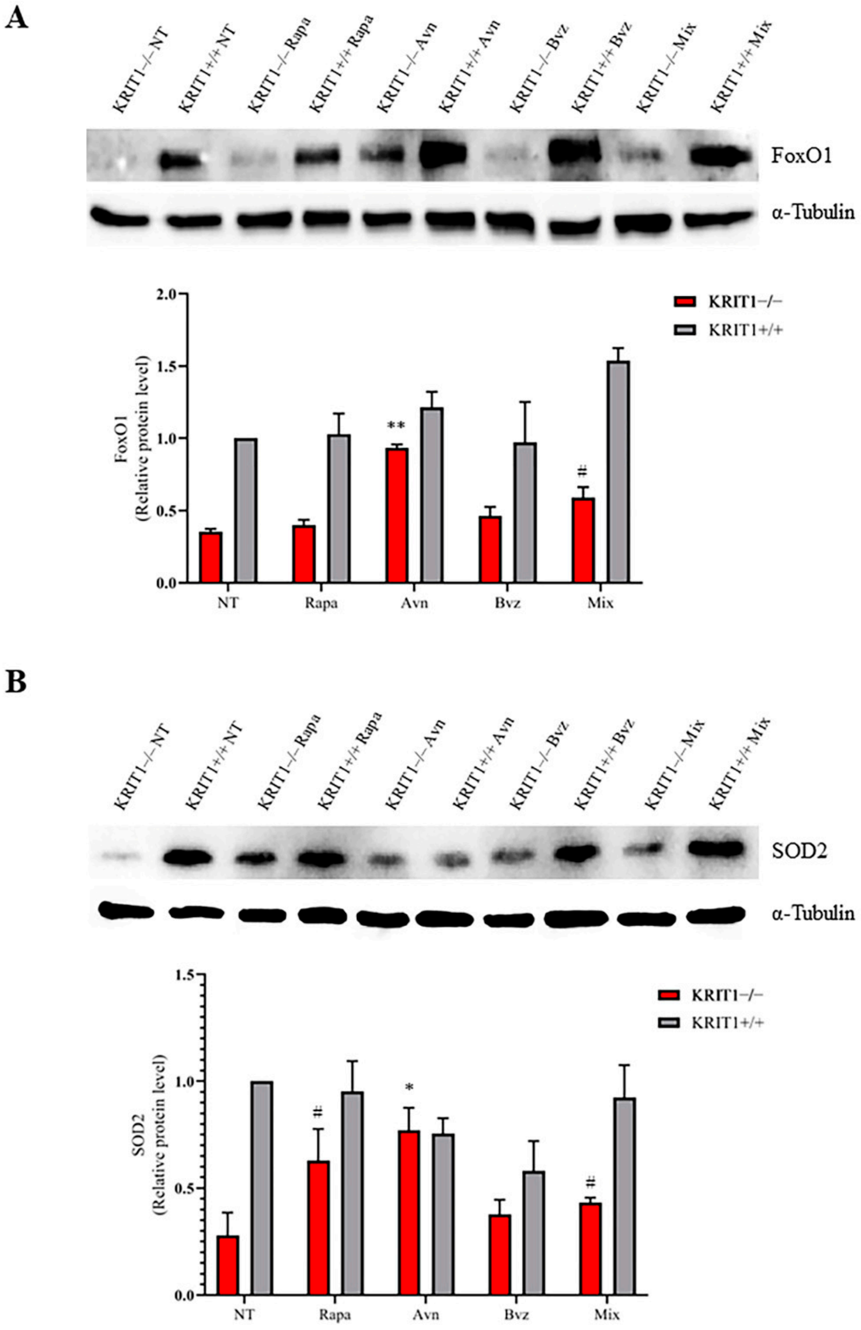

Figure S2. (A) Immunoblot analysis and representative histogram of the oxidative stress marker forkhead box class O1 (FoxO1) in KRIT1-knockout (KRIT1–/–) and KRIT1-overexpressing (KRIT1+/+) mouse embryonic fibroblasts (MEFs), untreated (Ctrl) or treated for 24 hours with free rapamycin (Rapa), bevacizumab (Bvz), yeast avenanthramide I (Avn), or with a combination of all three compounds (herein indicated as Mix) to a final concentration of: Avn = 15 μg/mL (50 μM), Rapa = 0.46 μg/mL (500 nM), Bvz = 10 μg/mL (70 nM). After treatment cells were lysed, as described in Materials and methods, and analysed for indicated protein by Western blot (WB) analysis. (B) Immunoblot analysis and histogram representing the quantitative evaluation by densitometric analysis of superoxide dismutase 2 (SOD2) protein expression levels. Quantifications are relative protein level units referred to average value obtained for KRIT1+/+ samples. α-tubulin was used as internal loading control for WB normalization. Statistical analysis: treatments vs NT in KRIT-/- MEFs: # $p<0.1$; *$p<0.05$; **$p>0.01$.

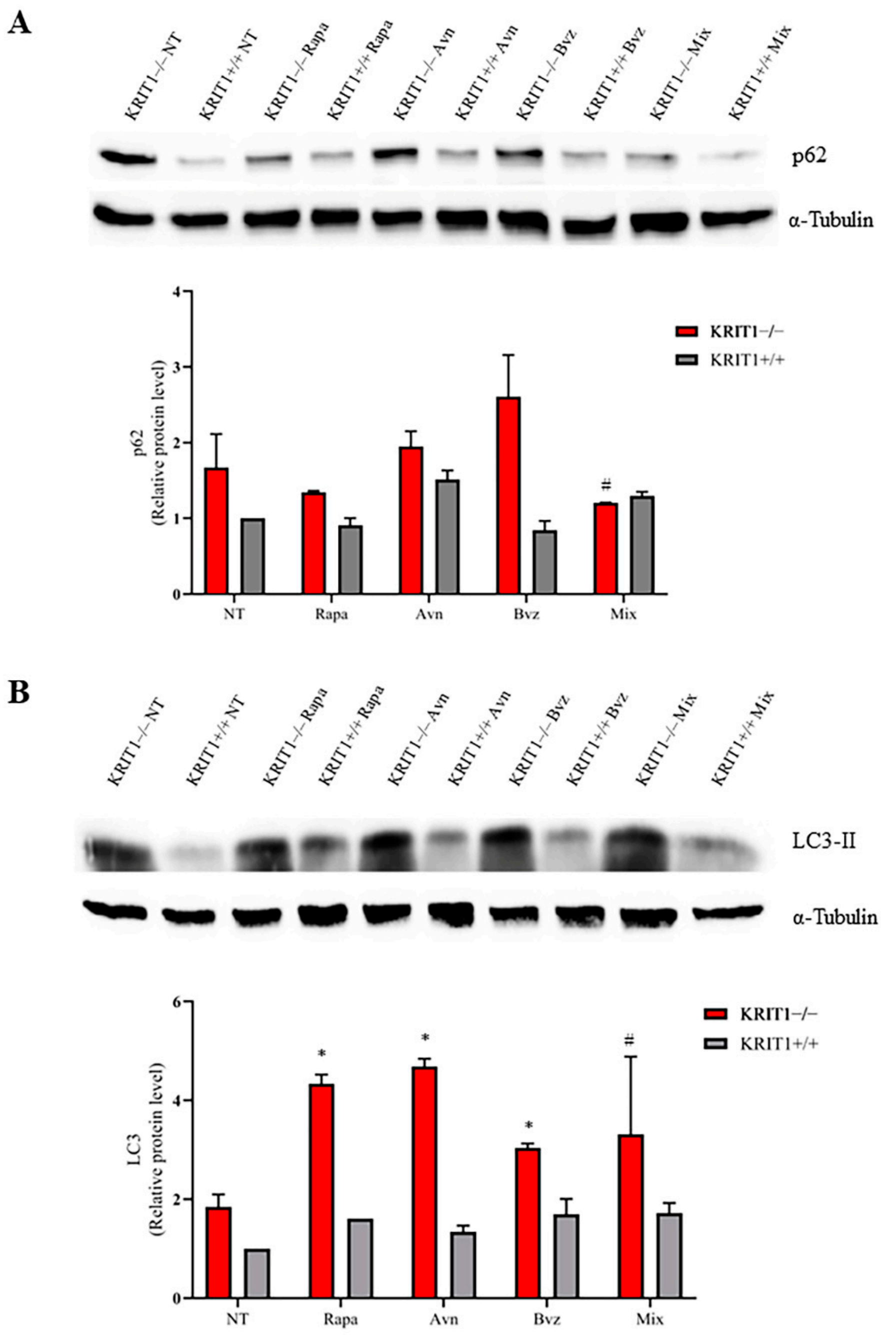

Figure S3. (A) Immunoblot analysis and representative histogram of the autophagic marker Sequestosome-1 (p62) in KRIT1-knockout (KRIT1–/–) and KRIT1-overexpressing (KRIT1+/+) mouse embryonic fibroblasts (MEFs), untreated (Ctrl) or treated for 24 hours with free rapamycin (Rapa), bevacizumab (Bvz), yeast avenanthramide I (Avn), or with a combination of all three compounds (herein indicated as Mix) to a final concentration of: Avn = 15 μg/mL (50 μM), Rapa = 0.46 μg/mL (500 nM), Bvz = 10 μg/mL (70 nM). After treatment cells were lysed, as described in Materials and methods, and analysed for indicated protein by Western blot (WB) analysis. (B) Immunoblot analysis and histogram representing the quantitative evaluation by densitometric analysis of light chain 3 (LC3) total protein expression levels. Quantifications are relative protein level units referred to average value obtained for KRIT1+/+ samples. α-tubulin was used as internal loading control for WB normalization. Statistical analysis: treatments vs NT in KRIT–/– MEFs: # $p<0.1$; *$p<0.05$.

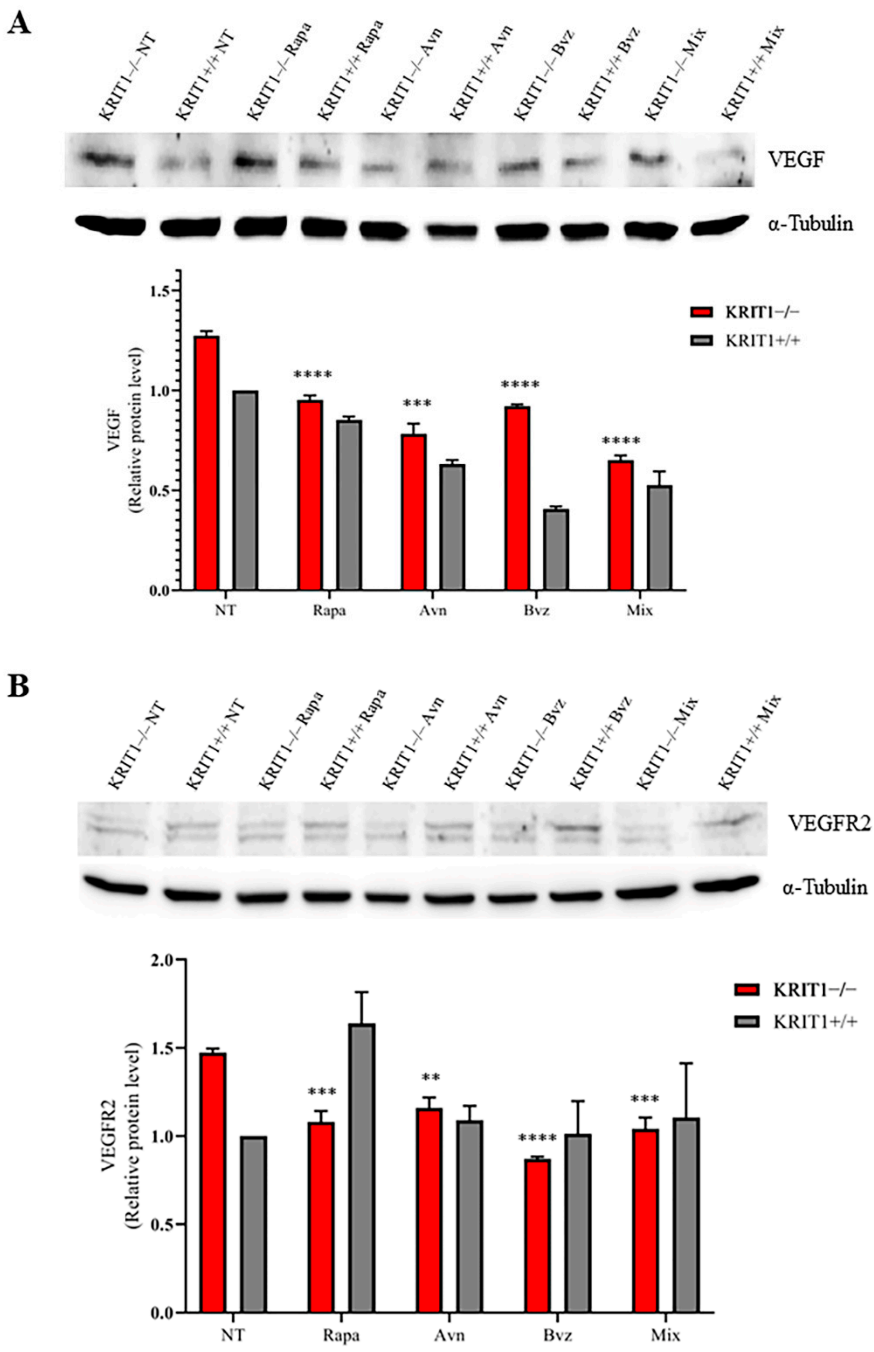

Figure S4. (A) Immunoblot analysis and representative histogram of vascular endothelial growth factor (VEGF) in KRIT1-knockout (KRIT1–/–) and KRIT1-overexpressing (KRIT1+/+) mouse embryonic fibroblasts (MEFs), untreated (Ctrl) or treated for 12 hours with free rapamycin (Rapa), bevacizumab (Bvz), yeast avenanthramide I (Avn), or with a Mixture of all three compounds (herein indicated as Mix) to a final concentration of: Avn = 15 μg/mL (50 μM), Rapa = 0.46 μg/mL (500 nM), Bvz = 10 μg/mL (70 nM). After treatment cells were lysed, as described in Materials and methods, and analysed for indicated protein by Western blot (WB) analysis. (B) Immunoblot analysis and histogram representing the quantitative evaluation by densitometric analysis of VEGFR2 protein expression levels. Quantifications are relative protein level units referred to average value obtained for KRIT1+/+ samples. α-tubulin was used as internal loading control for WB normalization. Statistical analysis: treatments vs NT in KRIT-/- MEFs: # p<0.1; *p<0.05; **p>0.01; ***p<0.005; ****p<0.0001.
